# Mortality in Patients with Diabetic Foot Ulcers: Causes, Risk Factors, and Their Association with Evolution and Severity of Ulcer

**DOI:** 10.3390/jcm9093009

**Published:** 2020-09-18

**Authors:** José Antonio Rubio, Sara Jiménez, José Luis Lázaro-Martínez

**Affiliations:** 1 Diabetic Foot Unit, Department of Endocrinology, Hospital Universitario Príncipe de Asturias, Alcalá de Henares, 28805 Madrid, Spain; sjimenezg@salud.madrid.org; 2 Department of Biomedical Sciences, Universidad de Alcalá, Alcalá de Henares, 28805 Madrid, Spain; 3 Diabetic Foot Unit, Universidad Complutense de Madrid, Instituto de Investigación Sanitaria del Hospital Clínico San Carlos (IdISSC), 28040 Madrid, Spain; diabetes@ucm.es

**Keywords:** diabetic foot ulcers, ulcer severity, morbidity, mortality, diabetic foot unit, reulceration, SINBAD system

## Abstract

**Background:** This study reviews the mortality of patients with diabetic foot ulcers (DFU) from the first consultation with a Multidisciplinary Diabetic Foot Team (MDFT) and analyzes the main cause of death, as well as the relevant clinical factors associated with survival. **Methods:** Data of 338 consecutive patients referred to the MDFT center for a new DFU during the 2008–2014 period were analyzed. Follow-up: until death or until 30 April 2020, for up to 12.2 years. **Results:** Clinical characteristics: median age was 71 years, 92.9% had type 2 diabetes, and about 50% had micro-macrovascular complications. Ulcer characteristics: Wagner grade 1–2 (82.3%), ischemic (49.2%), and infected ulcers (56.2%). During follow-up, 201 patients died (59.5%), 110 (54.7%) due to cardiovascular disease. Kaplan—Meier curves estimated a reduction in survival of 60% with a 95% confidence interval (95% CI), (54.7–65.3) at 5 years. Cox regression analysis adjusted to a multivariate model showed the following associations with mortality, with hazard ratios (HRs) (95% CI): age, 1.07 (1.05–1.08); HbA1c value < 7% (53 mmol/mol), 1.43 (1.02–2.0); active smoking, 1.59 (1.02–2.47); ischemic heart or cerebrovascular disease, 1.55 (1.15–2.11); chronic kidney disease, 1.86 (1.37–2.53); and ulcer severity (SINBAD system) 1.12 (1.02–1.26). **Conclusion:** Patients with a history of DFU have high mortality. Two less known predictors of mortality were identified: HbA1c value < 7% (53 mmol/mol) and ulcer severity.

## 1. Introduction

Approximately one in four patients with diabetes will develop a foot ulcer in their lifetime [1]. Seventy percent of diabetic foot ulcers (DFU) remain unhealed after 20 weeks of treatment [2], and 60% of them become infected, and of these, 20% end in different levels of amputation [3].

Avoiding unnecessary amputations is the primary goal of managing diabetic foot disease (DFD), and most interventions for managing these patients focus on the restoration of tissue perfusion and infection control [4]. Current clinical guidelines have developed a great deal of information on preventive and therapeutic interventions aimed at reducing the sequence of events (foot at risk, lesion, and subsequent amputation); but how other aspects, such as patient mortality and certain clinical factors, glycemic control, ulcer evolution and severity influence patient survival are less well known [5,6].

Patients with DFU have an increased risk of all-cause mortality, which is estimated to be more than double that of patients with diabetes without this complication [7]. On average, a person who develops DFU has a 3–5 year lower survival rate than a counterpart with diabetes, and this effect is in addition to that associated with diabetes itself, for which the reduction in life expectancy is about 6 years [8,9]. Survival is reduced by 40% in 5 years [10,11], although it is further reduced for patients with more severe lesions [12] or with ischemic versus non-ischemic lesions [13]; we have little data regarding the long-term survival of diabetic patients with this complication [7].

Cardiovascular disease is the main cause of mortality among patients with diabetes (50–60%) [14]. However, this aspect is less well known among patients with DFD. It has been reported that the rate of deaths by cardiovascular disease in patients with DFD, ranges from 50–70% [9,15] to 20% [8,16], probably associated with the inconsistency and variability of the studies’ data.

Ulcer severity has been associated with the worst patient outcomes and a higher rate of lower limb amputation. However, there are few studies that have investigated the association between ulcer severity and the rate of mortality [13,17,18]. Knowing the main causes of death of patients with DFD and the clinical factors associated with the risk of mortality could be useful in the implementation of therapies and interventions that could reduce this burden. A multidisciplinary approach to DFD has been demonstrated to be the most effective strategy to reduce the rate of amputation and mortality [19]. Managing the patient as a whole, allows better control of diabetes and its co-morbidities and could help in obtaining better patient outcomes. In 2008, a specialized diabetic foot unit was set up at a tertiary hospital in Spain led by an endocrinologist and a podiatrist, working with a multidisciplinary diabetic foot team (MDFT) involving different specialties: vascular surgery, general surgery, vascular and interventional radiology, orthopedic surgery, infectious diseases, and physical medicine and rehabilitation [20]. We aimed to describe the rate of mortality among patients with DFD from the first consultation at our unit and analyze the main cause of death, as well as the relevant clinical factors associated with their survival.

## 2. Materials and Methods

We included in the study all consecutive patients who were referred to our MDFT center with a new DFU between 1 February 2008 and 31 December 2014. Patients came from the area of influence of our hospital, covering a large town (Alcalá de Henares) in the south of Madrid (Spain) and 12 nearby villages. Patients were referred either by Primary Care or specialized care centers and by the hospital’s emergency gate. All patients were followed up until death or the last date for which data could be obtained from the electronic case history. Subjects with less than one year of follow-up were excluded. The last registration date was 30 April 2020.

### 2.1. Description of the Multidisciplinary Diabetic Foot Team

The functioning of the MDFT is described in greater detail in previous articles [21,22]. Diagnostic and management approaches were decided following the International Diabetic Foot Consensus guidelines [5], and the coordination with other specialties was established as required. If patients have more severe lesions, they are mainly referred to the general and vascular surgery departments for hospital admissions or outpatient visits. Regardless of whether hospital admission or assessment by other specialties was required or not, all patients were monitored at the diabetic foot unit until the end of the episode, to optimize and coordinate the control of blood glucose and co-morbidities. Once the lesion had healed, frequent follow-up visits by the MDFT were scheduled according to the risk of reulceration [5].

### 2.2. Data Collection and Processing

Patients’ clinical characteristics were collected from a database specifically designed for patient follow-up at the MDFT. Information regarding follow-up was also collected from the HORUS platform in order to improve the compilation of the variables and to more accurately assess current patient status. This platform allows access to the Primary Care electronic case history, as well as to the reports of the hospitals of the Madrid Regional Health Service (SERMAS) and is shared by the entire Madrid Region.

We defined the study variables as follows: (I) Chronic kidney disease (CKD) was defined by the presence of urine albumin/creatinine ratio of ≥ 30 mg/g (at least two measurements), or the glomerular filtration rate (GFR) (estimated from the MDRD-4 equation) < 60 mL/min. (II) Sensory neuropathy was defined as the absence of sensitivity with monofilament (10 g) or tuning fork (64–128 Hz). If there were multiple lesions, only the most severe was registered. (III) Ischemic lesion was defined as the absence of distal pulses or confirmatory diagnostic tests: The ankle-brachial index < 0.9, the toe-brachial index < 0.6, or transcutaneous oxygen pressure < 30 mmHg. (IV) Minor amputation was defined as amputation distal to the ankle joint, while major amputation was defined as amputation through or proximal to the ankle joint. (V) Reulceration was defined as a new full-thickness lesion of the skin on patients’ feet that occurred after the first DFU was healed (ulcer healed was defined as 100% epithelialization without exudate, confirmed at least 4 weeks after closure was first assessed). The severity of ulceration was scored according to the Wagner staging system (1–5) and SINBAD score [23]. The severity of infection was graded according to IWGDF/IDSA criteria (0–3) [23].

The main cause of death was obtained from the clinical reports during hospital admission, or alternatively from the Primary Care electronic case history. If the death occurred unexpectedly outside the hospital, the cause was registered as probably cardiovascular, and was grouped together with ischemic heart disease, heart failure, and cerebrovascular disease.

### 2.3. Data Reporting and Statistical Analysis

Quantitative data were expressed as the median and interquartile range (Q25–Q75), and qualitative data were reported as absolute values and percentages (%).

Univariate and multivariate Cox regression adjusted for independent variables in two models was used to determine which variables were associated with mortality. Multivariate analysis models were developed from the independent variables with a *p* < 0.05 and those of clinical interest: age, sex, years since diagnosis, type of diabetes mellitus (DM), active smoking, CKD, retinopathy, cardiovascular disease, history of amputation, and HbA1c. The variables were grouped into two models, 1 and 2. Hazard ratio (HR) (95% confidence interval (95% CI)) was used as a risk measure. Kaplan—Meier function and log-rank test were used to test the equality of survivor functions between the various groups and showed some variables of clinical interest for the present study. Survival analysis was estimated 5 years after the first evaluation at the MDFT. The log-rank test was obtained from the first 5 years of the survival analysis. Reulceration was analyzed in the subjects who survived after the first episode of ulceration and who had not required a major amputation. The SPSS version 19.0 statistical package was used (IBM, Armonk, NY, USA). Values of *p* < 0.05 were considered statistically significant.

### 2.4. Ethical Issues

The study was approved by the Clinical Research Ethics Committee of the HUPA (reference OE 26/2015). Since this was a retrospective, observational study, patient-informed consent was not requested. In some cases, patients were no longer followed up by the MDFT or had died before the start of the study. Patient data were anonymized to preserve confidentiality.

## 3. Results

Of the 345 patients who consulted the unit, 338 were included (Table 1). Seven patients were excluded because the follow-up period was less than 1 year. Median age at first presentation of diabetes mellitus patients (DM) was 71 years (62–80), 65.4% males and 97% European ethnicity. Median follow-up over the study period was 8 years (6.2–9.5), with a range of 1.2–12.2 years. The majority (92.9%) had type 2 DM and many years of evolution (median 14). Most patients (59.2%) required insulin treatment, and half of them had chronic complications: retinopathy (57.4%), chronic kidney disease (43.8%), and cardiovascular disease (47.3%). The metabolic control was poor, and 50% of patients had HbA1c values > 7.9% (63 mmol/mol).

The majority (82.3%) of the ulcers were Wagner grade 1–2, half of the patients (49.2%) had ischemic lesions, and 56.2% showed some degree of infection: 111 (32.8%), 65 (19.2%), and 14 (4.1%) grade 1–3 respectively, according to IWGDF/ISDA criteria. The SINBAD system score was 3 (2–4). A history of DFU (41.1%) and amputation (15.7%) was common at the first consultation. Of the 338 patients, 257 (76%) achieved healing of the lesion, 39 (11.5%) required minor amputation, 24 (7.1%) required major amputation, and 18 (5.3%) died with unhealed lesions. The median healing time was 4.5 weeks (2–10).

During the follow-up period, 201 patients died (59.5%), and 139 deaths (69.2%) occurred during hospitalization. Table 2 shows the main causes of death, with cardiovascular disease being the most common cause of mortality (54.7%), followed by respiratory disease (18.9%). In 11 patients (5.5%), DFU was identified as the cause of death.

**Figure 1:**
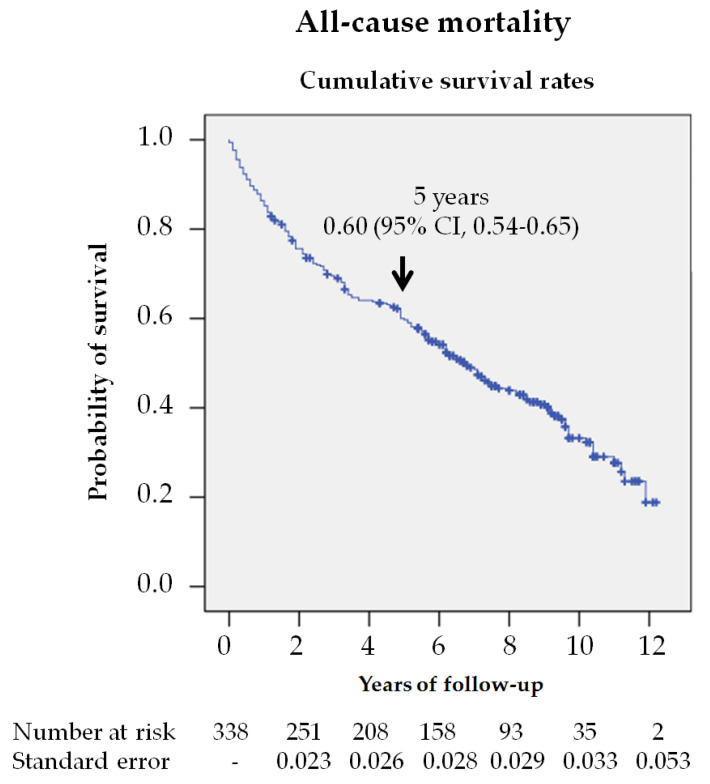
Survival analysis of the 338 patients with diabetic foot ulcers (DFU) from their first consultation at the MDFT center. The Kaplan—Meier curve shows the estimated 5-year survival rate.

Figure 1 shows the survival rate of the 338 patients using the Kaplan—Meier curve, estimating a reduction in survival of 60% (95% CI, 54.7–65.3) at 5 years.

**Figure 2:**
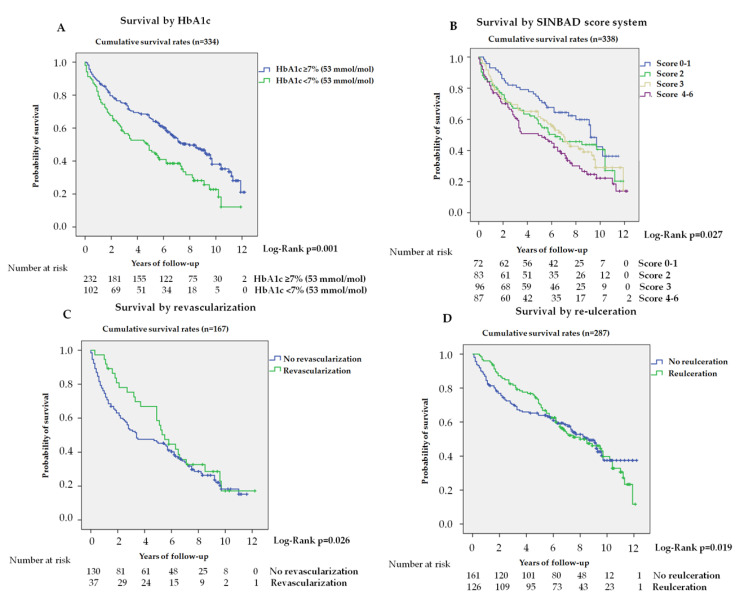
**Kaplan**—**Meier curves for survival.** (**A**): Survival by HbA1c in total cohort (n = 334, 4 subjects no data). (**B**): Survival by SINBAD score system (n = 338). (**C**): Survival by revascularization in patients with ischemic lesion (n = 167). (**D**): Survival by reulceration in patients after the resolution of the DFU and without major amputation (n = 287).

Table 3 shows the variables predicting for survival, analyzed by univariate and multivariate Cox regression. In univariate analysis, the main contributors to increased mortality were: age; type 2 diabetes mellitus (T2DM); years of evolution; low HbA1c; ischemic heart or cerebrovascular disease; CKD; reduction of glomerular filtration; history of major amputation or peripheral artery disease; ischemic etiology, ulcer and lesion severity (SINBAD system score). Other variables not shown, such as Wagner’s degree or degree of infection, were not significant as predictors of mortality. In multivariate analysis, adjusted to models 1 and 2, some variables proved to be significant and, therefore, independent variables predicting mortality: age; HbA1c < 7% (53 mmol/mol); current smoker; ischemic heart or cerebrovascular disease; CKD; reduction of glomerular filtration; and lesion severity. Kaplan—Meier curves for survival by HbA1c and by the SINBAD score system are shown in Figure 2A,B, respectively.

Of the 167 patients with an ischemic lesion, 37 (22.2%) required revascularization procedures (22—surgical bypass, 11—endovascular techniques, and 4—combined treatment). Kaplan—Meier curves for survival in patients who underwent revascularization are shown in Figure 2C. The log-rank test proved to be significant (*p* = 0.026), at 5 years. During the first 5 years, in univariate analysis, revascularization was associated with lower mortality, HR 0.50 (95% CI 0.27–0.94), but in the multivariate analysis, it did not remain significant: HR 0.87 (95% CI 0.46–1.67) in model 1 and HR 0.77 (95% CI 0.38–1.55) in model 2.

In the reulceration sub-study, we included 287 patients after the resolution of the first DFU episode. Eighteen patients who died with the lesion and 33 who had a major amputation were excluded. During the follow-up, 126 patients (43.9%) suffered a new ulcer event. The time to reulceration was 0.9 years (0.43–1.75). Kaplan—Meier curves for survival by reulceration are shown in Figure 2D. The log-rank test was significant during the first 5 years of follow up (*p* = 0.019). During these first 5 years, the univariate analysis by Cox regression showed an HR 0.58 (95% CI 0.37–0.92), but lost significance in the analysis adjusted to model 1, HR 0.73 (95% CI 0.44–1.17) and model 2, HR 0.72 (95% CI 0.43–1.19).

## 4. Discussion

### 4.1. Global Mortality

In this study, almost 60% of the patients died during the follow up, which indicates that survival was reduced to 60% at 5 years. Jupiter et al. [10] published a systematic review, which included 12 original studies, finding a 5-year mortality rate of about 40% in patients with a diabetic foot ulcer. However, there are fewer studies investigating longer-term mortality. Morbach et al. [17] and Mader et al. [15] found 10-year mortality rates like those found in our study, 70% and 64%, but Iversen et al. [24] and Jeyaraman et al. [16] reported lower rates, 49% and 45%, respectively. These differences can be explained by the fact that in studies with lower mortality rates, younger subjects, with fewer years of evolution and with less macrovascular disease were included.

### 4.2. Cardiovascular Disease as a Cause of Death

In agreement with other reports in the literature [9,18,24], the main cause of death in our population was cardiovascular disease, followed in order of frequency by respiratory disease. This last observation is reasonable considering the previous or current high exposure to tobacco in our series (50.2%) and the high prevalence of ischemic heart disease or cerebrovascular disease (47.3%). Other studies have found lower mortality rates from cardiovascular disease, close to 20% [8,16]. Methodological aspects, such as data collection from death certificates or clinical databases, might explain these differences [25]. In postmortem studies, the lack of difference in causes of death between patients with diabetes, with and without DFD [9], as well as the high frequency of death due to cardiovascular disease in cardiovascular safety studies [26], indicate that cardiovascular disease is the leading cause of death in diabetic patients. Considering that the mortality of diabetic patients with an established cardiovascular disease does not exceed 3% per year [27,28], patients with a history of DFU are at much greater risk, resulting in patients at extreme cardiovascular risk [29]. All these data suggest that patients with DFD should be treated proactively, intensifying control of cardiovascular risk factors.

It is not well understood why these patients have a high mortality rate. Perhaps the most relevant hypothesis is that these patients have different co-morbidities, such as CKD, high rate of micro- and macro-vascular disease, and (especially) the presence of peripheral neuropathy [9]. In this study, we did not find an association between mortality and neuropathy, assessed by loss of protective sensitivity (monofilament or tuning fork), as in the study by Amadou et al. [30]. Perhaps methodological aspects in the assessment of peripheral neuropathy could explain these differences [31]. However, peripheral neuropathy and autonomic cardiovascular neuropathy often coexist in the same patient, a combination that results in increased myocardial ischemia as either silent ischemia or with a poorer adaptive response to ischemic insults [32,33]. The higher frequency of ischemic heart disease as a cause of death among neuropathic versus ischemic patients supports these data [9].

### 4.3. Diabetic Foot Ulcer as a Cause of Death

The analysis of the causes of mortality also showed that in 11 patients (5.5%), the lesion was identified as the mortality-triggering factor in the hospital clinical reports, mainly due to sepsis and consecutive multiple organ failure. Morbach et al. [17] reported that up to 5.9% of patients with DFU died from the lesion, without specifying whether the ulcer was the cause of death; while Ghanassia et al. [34] reported that of 19.6% of patients hospitalized for the ulcer, the lesion was the cause of death. However, this outcome is less well-reported and controversial and requires further research; it is important to take it into account in the possible evolution and management of these patients.

### 4.4. Predictive Factors for Mortality

Univariate and multivariate analysis adjusted to different variables (models 1 and 2) showed that some factors independently predicted mortality in the population with DFU. Most factors, such as age, smoking, ischemic heart disease, cerebrovascular disease, and renal disease, have been documented in previous studies [10,17,24] and are non-controversial, and can be seen in any patient with diabetes. One discordant aspect was the non-association of male sex with mortality in our study, as reported by other authors [18,35], suggesting that in the presence of DFD, the mortality rate is similar in both sexes. Seghieri et al. [35] found even higher mortality in women versus men in non-vascular DFD.

#### 4.4.1. Glycemic Control

Glycemic control is less well known and of greater practical interest. In our study, tighter glycemic control estimated by HbA1c independently predicted higher mortality. Thus, patients with HbA1c < 7% (53 mmol/mol) had a HR of 1.43 (95% CI 1.02–2.0) when multivariate analysis was adjusted to model 2. From a practical perspective, this means that a patient with DFD without ischemic heart disease and HbA1c < 7% (53 mmol/mol) has a similar risk of death as another patient also with DFD, but without ischemic heart disease and a HbA1c value of 8% (64 mmol/mol).

Studies examining this aspect found no association [12,36,37] or found a similar association to our study: increased HbA1c and fewer deaths [11,16,18]. Only the study by Mader et al. [15] found a worse glycemic control association with more mortality. These results, although initially contradictory, agree with what has been published in intervention studies in the diabetic population (T1DM and T2DM) in the scenario of presenting many years of evolution and the great burden of micro- and macro-vascular complications [38]. Aspects such as the increased risk of hypoglycemia in patients with tighter control could explain this higher mortality in patients at high cardiovascular risk [39]. Recently, a secondary analysis of the TECOS Randomized Clinical Trial, supported these data [40]. The aforementioned suggests that in patients with a history of DFU, very tight glycemic control with HbA1c < 7% (53 mmol/mol) is not recommended and that aiming for improved control by reducing HbA1c values is unlikely to reduce mortality in the short-medium term, considering the shorter life expectancy of patients with this chronic complication. An adequate degree of glycemic control—taking account of age, co-morbidity, and life expectancy—is mandatory.

#### 4.4.2. Ulcer Severity

Few studies analyze the association between mortality and severity of ulcers; however, there are more studies that analyze the association with its etiology, increasing mortality among patients with ischemic lesions [13,17,18]. In our series, the SINBAD score system was independently associated with mortality, HR 1.14 (95% CI 1.01–1.27) and HR 1.12 (95% CI 1.02–1.26) adjusted to models 1 and 2, respectively. Winkley et al. [18], using the Texas classification, found no association with mortality; but recently, Brennan et al. [12] using a simple classification, gangrene and osteomyelitis versus early ulcer, as well as Amadou et al. [30], using the PEDIS classification, have reported this association. It should be noted that the classification used by Amadou et al. only considered perfusion.

The SINBAD score is recommended by the IWGDF for communication among health professionals and includes different prognostic factors of the lesion such as location, perfusion, neuropathy, infection, and depth, allowing a detailed description of the lesion and evaluation of many clinical factors [23]. This study, therefore, demonstrates that the ulcer severity estimated by this classification system is a predictor of death, independent of systemic factors and should be a guide for decisions in day-to-day practice.

#### 4.4.3. Revascularization

In patients with DFU, the presence of chronic limb-threatening ischemia is associated with increased mortality, lower limb amputation, and loss of quality of life [41]. In our series, 22.2% of patients with ischemic lesions received revascularization treatment, observing a longer survival during the first 5 years. Analysis during the first 5 years showed a 50% reduction in mortality in the treated group; however, these differences were not maintained when adjusted for other variables. Aspects such as patient selection based on functional status, complex and unfavorable anatomy, and co-morbidity might explain these differences [42].

#### 4.4.4. Reulceration

Reulceration is common in diabetic patients following DFU [43], but its association with mortality is poorly known. In this study, 43.9% of patients after healing suffered reulceration, with lower mortality, HR 0.58, during the first 5 years in the univariate analysis. This reduction was not observed when the entire follow-up period was analyzed, nor did it remain significant after adjusting for different variables, indicating that the association between reulceration and survival depended on age and co-morbidities. Winkley et al. [18] also observed in a series of 229 patients followed for 18 months after their first DFU that patients with reulceration had lower mortality, but the analysis was unadjusted, and no other variables were taken into account. These results could be explained by considering the relationship between activity level and ulceration in patients at risk [43] and that activity level is reduced in patients with greater fragility and co-morbidities [44]. In patients with DFD, where sarcopenia is more common [45], reulceration is a marker of functionality and better health, improving survival.

#### 4.4.5. Limitations and Strengths

Within the limitations, it should be noted: (i) causes of death were collected from clinical reports and electronic case histories, with no death certificates for those who died outside the hospital setting; (ii) all variable data were obtained at the beginning of the study, but we could not analyze how they changed during the follow-up period; and (iii) we do not have some variables for the analysis of data such as: antiplatelet and lipid-lowering medications and types of oral drugs for the control of hyperglycemia. The main strengths of the study were: (i) real-world clinical practice population; (ii) large sample size and long follow-up time (up to 12.2 years) allowing for medium-long term outcomes; and (iii) the data were obtained from a specially-designed database.

## 5. Conclusions

In the present study, patients with a history of diabetic foot ulcers had very limited survival; estimated survival was reduced to 60% at 5 years. The most common cause of death is cardiovascular disease. Within the predictive factors of mortality, besides the well-known factors, such as age, active smoking, cardiovascular disease, and renal disease, we also identified as independent factors of mortality, patients with a HbA1c value < 7% (53 mmol/mol) and those with more severe lesions. Therefore, there is evidence to support the idea that diabetic patients with a history of foot ulcers are at very high cardiovascular risk and should be treated by intensifying control of modifiable risk factors, preferably using drugs with proven reduction of cardiovascular events and deintensifying treatment in order to obtain the best possible glycemic control without significant hypoglycemia. The severity of the lesion should also be regarded as a prognostic marker of mortality.

## Figures and Tables

**Table 1:** Baseline characteristics at first outpatient presentation to the Multidisciplinary Diabetic Foot Team (MDFT) center (total cohort n = 338 subjects).

	Median (Q25–Q75)	Range
Age in years	71 (62–80)	32–95
Years since diagnosis	14 (8–23)	0–56
HbA1c (%) ^a^	7.9 (6.7–9.1)	4.1–13.7
HbA1c (mmol/mol) ^a^	63 (50–76)	21–126
BMI (Kg/m^2^)	27.7 (24.9–31.1)	18–50
	**N**	**%**
Type of DM		
T1DM	18	5.3
T2DM	314	92.9
Other types of DM	6	1.8
Sex		
Males	221	65.4
Females	117	34.6
Smoking		
Never	168	49.7
Former smoker	109	32.2
Current smoker	61	18.0
Alcohol intake (♀ > 25 g/day. ♂ > 40 g/day)		
Never	246	72.8
Former alcohol intake	51	15.1
Current alcohol intake	41	12.1
Treatment of hyperglycemia		
Dietetic treatment alone	17	5.0
Oral antidiabetic drugs or injections non-insulin	121	35.8
Insulin alone or with other treatments for hyperglycemia	200	59.2
Past ulcer	139	41.1
History of peripheral artery disease	109	32.2
Past amputation	*53*	15.6
Major	*15*	4.4
Minor	*38*	11.2
Retinopathy	194	57.4
Chronic kidney disease	148	43.8
Glomerular filtration rate (GFR)		
GFR > 60 mL/min	252	74.6
GFR 60–30 mL/min	57	16.9
GFR < 30 mL/min	12	3.6
Dialysis	17	4.4
Post-transplantation	2	0.6
Hypertension	272	80.5
Ischemic heart disease or cerebrovascular disease	160	47.3
Ischemic heart disease	133	39.3
Cerebrovascular disease	56	16.6
Sensory neuropathy	254	75.1
Ischemic lesion	167	49.4

Type 2 diabetes mellitus (T2DM). Type 1 diabetes mellitus (T1DM). ^a^ Normal values 4.2–6% and 22–42 mmol/mol standardized according to National Glycohemoglobin Standardization Program (NGSP) and Internacional Federation of Clinical Chemistry (IFCC) respectively.

**Table 2:** Main causes of death.

	N	%
Cardiovascular disease	110	54.7
Ischemic heart disease	25	12.4
Heart failure	20	19
Cerebrovascular disease	8	4
Probable cardiovascular cause. Unexpected death occurring outside hospital	57	28.4
Respiratory disease with or without infection	38	18.9
Multiorgan failure or sepsis associated with DFU	11	5.5
Multiorgan failure or sepsis from other causes	14	7
End-stage chronic kidney disease	12	6
Cancer	12	6
Other	4	2

**Table 3:** Independent association with all-cause mortality in univariate and multivariate analysis in patients with DFU.

	Unadjusted		Adjusted Model 1		Adjusted Model 2	
	HR (95% CI)	*p*-Value	HR (95% CI)	*p*-Value	HR (95% CI)	*p*-Value
Age	1.06 (1.04–1.07) *	<0.001	1.07 (1.05–1.09) *	<0.001	1.07 (1.05–1.08) *	<0.001
Male vs. female	1.09 (0.81–1.46)	0.544	1.27 (0.92–1.74)	0.135	1.28 (0.92–1.77)	0.140
T2DM vs. T1DM	2.47 (1.16–5.26) *	0.019	0.92 (0.38–2.21)	0.866	0.97 (0.40–2.35)	0.951
Years since diagnosis	1.01 (1.00–1.02) *	0.029	1.00 (0.99–1.01)	0.435	1.00 (0.99–1.02)	0.228
HbA1c (%) at first consultation	0.89 (0.82–0.97) *	0.008	0.95 (0.87–1.03)	0.262	0.93 (0.85–1.02)	0.175
HbA1c < 7 (%)/53 (mmol/mol) vs. ≥ 7 (%)/53 (mmol/mol)	1.71 (1.27–2.29) *	<0.001	1.37 (0.99–1.90)	0.056	1.43 (1.02–2.0) *	0.035
BMI (Kg/m2)	0.97 (0.94–1.00)	0.121	0.96 (0.93–1.00)	0.05	0.97 (0.94–1.01)	0.152
Current smoker	0.80 (0.55–1.16)	0.244	1.54 (1.01–2.32) *	0.04	1.59 (1.02–2.47) *	0.038
Ischemic heart disease or cerebrovascular disease	2.10 (1.58–2.78) *	<0.001	1.70 (1.27–2.27) *	0.04	1.55 (1.15–2.11) *	0.004
Ischemic heart disease	1.91 (1.45–2.53) *	<0.001	1.49 (1.11–1.98) *	0.006	1.35 (1.0–1.82) *	0.049
Cerebrovascular disease	1.38 (0.97–1.97)	0.07	1.09 (0.75–1.57)	0.635	1.11 (0.76–1.64)	0.569
Hypertension	1.55 (1.04–2.29)	0.029	1.08 (0.71–1.83)	0.708	1.15 (0.75–1.78)	0.507
Chronic kidney disease	1.68 (1.27–2.21) *	<0.001	1.73 (1.3–2.32) *	<0.001	1.86 (1.37–2.53) *	<0.001
Grouped GFR (1 = >60 mL/min, 2 = 30–60 mL/min, and 3 = <30 mL/min or on dialysis)	1.62 (1.32–1.98) *	<0.001	1.47 (1.19–1.82) *	<0.001	1.51 (1.21–1.89) *	<0.001
Retinopathy	0.80 (0.59–1.07)	0.8	0.82 (0.59–1.13)	0.232	0.82 (0.59–1.14)	0.255
Past amputation	1.32 (0.92–1.89)	0.125	1.26 (0.92–1.73)	0.137	1.35 (0.89–2.05)	0.152
Major	2.36 (1.31–4.25) *	0.004	1.89 (1.04–3.45) *	0.037	1.48 (0.75–2.90)	0.252
Minor	1.04 (0.68–1.59)	0.825	1.41 (0.91–2.19)	0.118	1.23(0.76–2.00)	0.385
History of peripheral artery disease	1.47 (1.15–1.96) *	0.007	0.99 (0.72–1.37)	0.973	0.96 (0.68–1.35	0.817
Sensory neuropathy ^a^	0.75 (0.55–1.02)	0.068	0.88 (0.64–1.23)	0.48	0.75 (0.53–1.07)	0.758
Ischemic lesion ^b^	2.2 (1.65–2.93) *	<0.001	1.27 (0.93–1.74)	0.128	1.25 (0.90–1.73)	0.182
Score SINBAD system	1.19 (1.08–1.32) *	<0.001	1.14 (1.01–1.27) *	0.023	1.12 (1.02–1.26) *	0.046

* Statistically significant variables. Type 2 diabetes mellitus (T2DM). Type 1 diabetes mellitus (T1DM). GFR: Glomerular filtration rate. ^a^ Sensory neuropathy, defined as the absence of sensitivity with monofilament (10 g) or tuning fork (64–128 Hz). ^b^ Ischemic lesion, defined as the absence of distal pulses or confirmatory diagnostic tests: the ankle-brachial index < 0.9, the toe-brachial index < 0.6, or transcutaneous oxygen pressure < 30 mmHg. Model 1: age, sex, years since diagnosis, current smoker, history of ischemic heart disease or cerebrovascular disease, chronic kidney disease, and past amputation. Model 2: model 1 + HbA1c, type of diabetes mellitus (DM), retinopathy, and the SINBAD system score.
